# 检测肺癌患者血清Cathepsin X及Cystatin C的临床意义

**DOI:** 10.3779/j.issn.1009-3419.2013.08.04

**Published:** 2013-08-20

**Authors:** 学德 张, 彦丽 侯, 泽群 牛, 维 李, 夏 孟, 娜 张, 拴盈 杨

**Affiliations:** 710004 西安，西安交通大学第二附属医院呼吸科 Department of Respiratory Medicine, the Second Afliated Hospital, Medical School of Xi'an Jiaotong University, Xi'an 710004, China

**Keywords:** Cathepsin X, Cystatin C, 肺肿瘤, 血清, Cathepsin X, Cystatin C, Lung neoplasms, Sera

## Abstract

**背景与目的:**

组织蛋白酶X（Cathepsin X, Cat X）是最近发现的一种组织蛋白酶（Cathepsins, Cats）家族成员。近年来研究表明Cat X与多种恶性肿瘤发生、发展有关。本研究旨在探讨肺癌患者血清Cat X及cystatin C的表达与临床特征及预后的关系。

**方法:**

采用ELISA法定量检测84例肺癌患者及36例健康对照者血清Cat X及cystatin C表达。

**结果:**

肺癌患者血清Cat X和cystatin C水平明显高于健康人（*P* < 0.01）；Cat X水平与肺癌病理类型之间有相关的趋势（*P*=0.076）。血清cystatin C水平与肺癌TNM分期正相关（*P*=0.01），cystatin C/Cat X与淋巴结转移之间有相关趋势（*P*=0.058）。Cat X表达水平与肺癌患者总生存期（overall survival, OS）相关，高水平Cat X肺癌患者OS更短。*Cox*单因素回归示Cat X高表达以及TNM分期是影响肺癌预后独立因素，*Cox*多因素回归显示，仅TNM分期是患者预后的独立危险因素。

**结论:**

肺癌患者中血清Cat X和cystatin C水平升高，检测肺癌患者Cat X和cystatin C血清水平对于指导临床肺癌诊断、评估预后有重要意义。

## Abstract (en)

**Background and objective:**

Cathepsin X (Cat X) has been identifed as a member of cathepsin family. Studies have shown that Cat X is involved in tumorigenesis and tumor development of various cancers. The aim of this study is to investigate the relationship between the clinicopathological prognosis and the levels of Cat X and cystatin C in the serum of patients with lung cancer.

**Methods:**

Blood samples were collected from 84 patients with lung cancer and 36 healthy control subjects. Cat X and cystatin C were determined by quantitative ELISA.

**Results:**

Cat X and cystatin C levels were signifcantly higher in the patients with lung cancer than that in the healthy control subjects (*P* < 0.01). Cat X level was correlated with the pathological types of lung cancer (*P*=0.076). Cystatin C was positively correlated with TNM stage (*P*=0.01). Furthermore, cystatin C/Cat X was correlated with lymph node metastasis (*P*=0.058). The patients with high Cat X levels experienced signifcantly shorter overall survival rates compared with those with low Cat X. Univariate analysis indicated that Cat X and TNM stage were related to overall survival. Multivariate *Cox* analysis indicated that TNM stage may be used as an independent prognostic variable in patients with lung cancer.

**Conclusion:**

Cat X and cystatin C levels were signifcantly higher in patients with lung cancer. Cat X and cystatin C detection in the sera may contribute to the diagnosis of lung cancer and may be used to evaluate the prognosis of patients with NSCLC.

组织蛋白酶(Cathepsins, Cats)属于溶酶体半胱氨酸蛋白酶，最初发现涉及到溶酶体内蛋白转换，近年来研究^[1, 2]^表明，还参与肿瘤的发生、发展、转移，有希望成为新的肿瘤诊断和预后判断标记物。Cat B、Cat L等在肺癌、乳腺癌、头颈癌及结肠癌等肿瘤中表达明显增高，且高表达的患者预后更差，对预测肿瘤的复发和预后有潜在价值^[3]^。Cat X是最近发现的一种Cats家族成员，它的结构和功能特点明显不同于其它Cats，有一个区别于其它Cats的非常短的原区(pro-region)和一个独特的迷你环(mini-loop)，Cat X不像其它Cats作为肽链内切酶发挥生物学作用，而靠水解羧基端氨基酸起作用。Cat X表达主要局限于各类免疫细胞中，如单核、巨噬细胞及树突状细胞^[4]^。它涉及到细胞间信号传导、细胞粘附、增生以及迁移等作用^[5-7]^。研究表明Cat X表达上调与幽门螺旋杆菌感染^[8]^、多发创伤^[9]^、肺结核^[10]^有关，也有报道发现Cat X在前列腺癌^[11]^、胃癌^[8]^、恶性黑色素瘤^[12]^中表达明显增高。此外，已经在多种肿瘤中发现染色体20q13中编码Cat X基因明显扩增^[13, 14]^。

Cystatin C是半胱氨酸蛋白酶抑制剂蛋白质中的一种，在所有的有核细胞内以恒定速度持续转录与表达，无组织特异性，并存在于各种体液之中，不受年龄、性别、体重、炎症等因素影响，是一种反映肾小球滤过情况的重要指标。目前研究^[15]^表明，它作为Cats抑制剂与Cats互相作用参与肿瘤发生、发展。近期研究发现血液、体液中cystatin C高表达的患者预后差，有希望作为预测肿瘤预后标记物。

目前为止，我们尚未发现肺癌患者血清Cat X的研究，cystatin C在肺癌患者血清研究的报道也少见，cystatin C作为Cats强有力的抑制剂，它与Cat X血清表达水平之间可能存在相关性。目前已有cystatin C与Cat B、Cat L的相关性研究，但是尚无cystatin C与Cat X之间的研究。本研究旨在检测肺癌患者和健康人血清Cat X与cystatin C表达水平以及二者相关性和与肺癌临床病理、预后关系。

## 1 材料与方法

### 1.1 研究对象

选择2007年3月-9月西安交大二附院84例肺癌患者，其中男性50例，女性34例，年龄41岁-78岁，中位年龄61.5岁；鳞癌30例，腺癌40例，小细胞肺癌(small cell lung cancer, SCLC) 14例。临床分期(TNM分期)，Ⅰ期+Ⅱ期28例，Ⅲ期+Ⅳ期56例。所有患者均经过组织学或病理学检查确诊为肺癌，采血前均未行手术、放化疗以及中成药等其它抗肿瘤治疗。另外，选取健康查体者36例作为对照组，男性20例，女性16例；年龄40岁-79岁，中位年龄60.4岁。本研究得到西安交大二附院伦理委员会许可，患者均签署知情同意书。

### 1.2 标本采集

所有肺癌患者和健康体检者分别抽取晨间空腹静脉血3 mL，抽取后1 h内，4 ℃，3, 000 g离心10 min，分离的血清-80 ℃冻存待检。

### 1.3 检测方法

Cat X及cystatin C水平均采用酶联免疫吸附法测定。严格按照ELISA检测试剂盒(上海郎卡公司)的说明书操作，标准品孔各加不同浓度的标准品50 μL；然后样本孔先加待测样本10 μL，再加样本稀释液40 μL；后分别加入辣根过氧化物酶(HRP)标记的检测抗体100 μL，封板膜封孔，37 ℃恒温箱温育60 min。手工洗板法清洗，每孔加入底物A、B各50 μL，37 ℃避光孵育15 min，后每孔加入终止液50 μL，15 min内，在450 nm波长处测定各孔的OD值。实验重复3次。

### 1.4 随访

84例患者术后均进行了随访，总生存期OS定义为首次确诊至患者死亡时间或末次随访时间。死于非肺癌相关原因的患者排除在本研究外。随访最长时间为60个月。

### 1.5 统计学处理

数据采用SPSS 16.0软件进行统计分析。每组数据均采用Mean±SD表示。组间差异采取*Mann-Whitney*和*Kruskal-Wallis*检验。*Pearson's*相关系数评价Cat X和cystatin C之间的相关性。*Kaplan-Meier*法(组间生存率比较采用*Log-rank*检验)及*Cox*单因素以及多因素风险回归模型进行生存分析。双侧*P* < 0.05为差异有统计学意义。

## 2 结果

### 2.1 肺癌患者和健康人Cat X和cystatin C表达水平比较

肺癌患者及健康人血清Cat X水平分别为(2.24±0.07) ng/mL、(1.85±0.09) ng/mL；cystatin C分别为(529.77 ±6.15) ng/mL、(476.76±13.95) ng/mL。肺癌患者血清Cat X和cystatin C水平明显高于健康人(*P* < 0.01)。此外，肺癌患者cystatin C/Cat X比值与健康人相比，差异无统计学意义(*P*=0.372)。*Pearson*相关分析显示，肺癌患者血清Cat X与cystatin C水平无明显相关性(*r*=-0.049, *P*=0.66)。

### 2.2 肺癌患者Cat X和cystatin C表达水平与临床病理特征的关系

Cat X和cystatin C/Cat X与肺癌患者性别、年龄、病理类型、淋巴结转移、细胞分化及TNM分期无明显相关，但Cat X与病理类型有相关趋势(*P*=0.076)，cystatin C/Cat X与淋巴结转移有相关趋势(*P*=0.058)。cystatin C水平与肺癌分期相关，Ⅲ期+Ⅳ期肺癌患者cystatin C水平明显高于Ⅰ期+Ⅱ期(*P*=0.01)，与其它临床病理特征无关(表 1)。

**1:** 肺癌患者血清Cat X和cystatin C表达水平与临床病理特征的关系 Relationship of Cat X and cystatin C in the sera of patients with lung cancer on the clinicopathological parameters

Characteristic		*n*	Cat X			Cystatin C			Cystatin C/Cat X	
			Mean±SD	*P*		Mean±SD	*P*		Mean±SD	*P*
Gender	Male	50	2.34±0.941	0.222		531.73±8.33	0.483		245.30±10.80	0.196
	Female	34	2.08±0.108			526.88±9.10			276.33±14.75	
Age (yr)	≥61.5	42	2.13±0.099	0.137		526.61±10.02	0.308		267.77±12.78	0.137
	< 61.6	42	2.34±0.103			532.92 ±7.22			247.95±12.29	
Pathological type	SCC	30	2.34± 0.125	0.076		537.14±9.53	0.575		251.45±15.42	0.076
	ADC	40	2.05 ±0.774			527.99±9.47			273.21±11.97	
	SCLC	14	2.56 ±0.233			519.05±15.03			227.69±22.92	
Lymph node metastasis	Yes	50	2.16±0.902	0.279		537.37±8.33	0.126		269.33±10.83	0.058
	No	34	2.38±0.117			516.09±8.03			237.21±14.94	
Grade	G1+G2	42	2.23±0.10	0.694		536.54±8.80	0.376		259.65±12.16	0.741
	G3	42	2.24±0.105			523.00±8.46			256.08±13.08	
Stage	Ⅰ+Ⅱ	28	2.22±0.148	0.232		507.99±8.94	0.01		258.23±17.72	0.992
	Ⅲ+Ⅳ	56	2.25± 0.08			540.66±7.71			257.68±10.07	
Cat X: Cathepsin X; Cats, Cathepsins; SCC: squamous cell carcinoma; ADC: adenocarcinoma; SCLC: small cell lung cancer.										

### 2.3 肺癌患者Cat X和cystatin C水平与预后的关系

#### 2.3.1 *Kaplan-Meier*生存分析

将患者性别、淋巴结状态、分化程度、分期分组，以及根据年龄、Cat X、cystatin C、cystatin C/Cat X的中位数以二分法的方式分 < 中位数组以及≥中位数组，进行*Kaplan-Meier*生存分析，生存差异的统计学意义用*Log-rank*法判定。结果示低Cat X和高Cat X患者中位OS分别为22.9个月和15.8个月，差异有统计学意义(*P*=0.019, RR=1.678)，低Cat X患者和高Cat X患者相比OS更长(图 1)。Ⅰ期+Ⅱ期和Ⅲ期+Ⅳ期肺癌患者中位OS分别为23.8个月和15.4个月，差异有统计学意义(*P*=0.011, RR=1.827)，晚期肺癌患者预后更差(图 2)。高、低cystatin C/Cat X值患者中位OS分别为23.5个月和18.5个月，差异无统计学意义，但有一定的差异趋势(*P*=0.069)。性别、年龄、淋巴结状态、分化程度以及cystatin C对肺癌患者OS无明显影响(表 2)。

**1:**
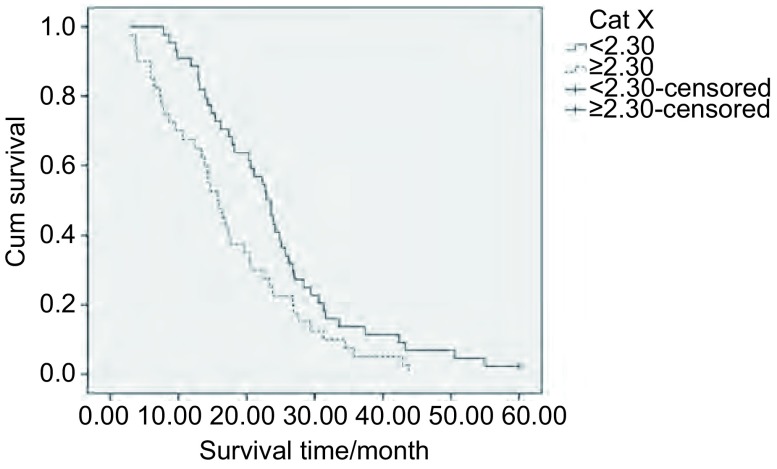
血清Cat X高表达组和低表达组肺癌患者*Kaplan-Meier*生存曲线 *Kaplan-Meier* survival curves of lung cancer patients with low or high levels of Cat X in sera

**2:**
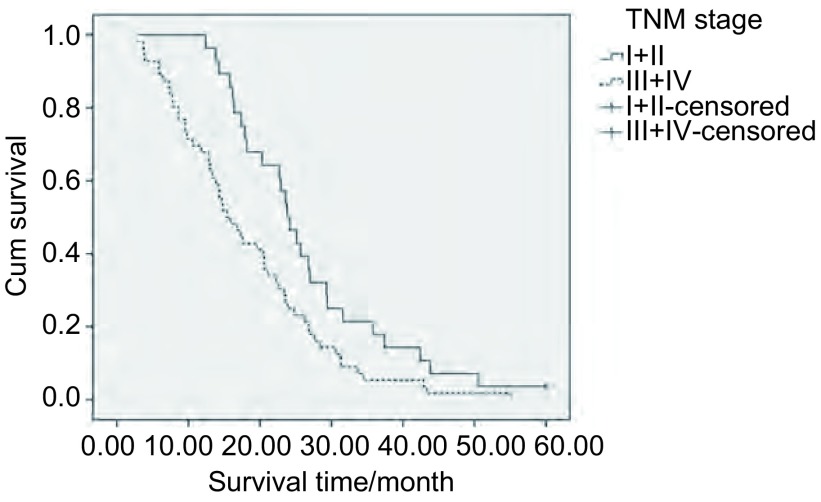
TNM Ⅰ+Ⅱ组和Ⅲ+Ⅳ组肺癌患者生存曲线 *Kaplan-Meier* survival curves of lung cancer patients with TNM stage Ⅰ+Ⅱ or stage Ⅲ+Ⅳ

**2:** 单因素和多因素分析肺癌Cat X、cystatin C及其它因素与预后的情况 Univariate and multivariate analysis of Cat X, cystatin C, and other potential factors for prognosis in patients with lung cancer

Characteristic		*n*	Overall survival (month)	Univariate analysis			Multivariate analysis	
				*P*	RR		*P*	RR
Cat X	< 2.30	44	22.9	0.019	1.678		0.768	1.131
	≥2.30	30	15.8					
Cystatin C	< 523.18	45	22.6	0.105	1.436		0.152	0.89
	≥523.18	39	17.3					
Cystatin C/Cat X	< 233.00	42	18.5	0.069	0.665		0.582	0.783
	≥233.00	42	23.5					
Gender	Male	50	17.3	0.103	0.688		0.358	0.789
	Female	34	22.3					
Age (yr)	< 61.5	42	21.1	0.177	1.357		0.376	1.26
	≥61.5	42	17.9					
Pathological type	SCC	30	17.6	0.638	1.078		0.193	1.25
	ADC	40	20.5					
	SCLC	14	17.9					
Lymph node metastasis	Yes	54	18.2	0.409	0.827		0.994	1.002
	No	30	20.3					
Grade	G1+G2	42	20.6	0.345	1.232		0.072	1.634
	G3	42	16.8					
Stage	Ⅰ+Ⅱ	28	23.8	0.011	1.827		0.009	2.25
	Ⅲ+Ⅳ	56	15.4					

#### 2.3.2 *Cox*比例风险回归模型分析

采取单因素及多因素*Cox*回归法分析影响肺癌患者预后的独立危险因素。利用*Cox*单因素回归分析发现Cat x高表达以及tnm分期是影响肺癌预后独立因素，继续将各个变量引入Cox多因素回归模型，结果显示，仅有TNM分期是患者预后的独立危险因素(表 2)。

## 3 讨论

肺癌是目前发病率高的恶性肿瘤之一，大部分发现时已经是中晚期，失去了手术机会，患者生存期短，5年生存率约15%^[16]^。因此肺癌的早诊断、早治疗至关重要。血清肿瘤标记物具有取材方便、方法易行等独特优势，在肺癌早期诊断、预后判断中有重要价值，因此人们一直在寻找有价值的血清肿瘤标记物。

Cat X是近年来发现一种新的Cats，通常以酶原形式存在于免疫细胞的溶酶体内，受到激活后，转化为有活性的酶释放到血液和细胞外基质中^[17]^。Cat X在恶性肿瘤患者血清和细胞外基质中也有表达，Vizin等^[18]^研究了Cat X在结肠癌、腺瘤、其它结肠良性病变以及健康者血清中的表达情况，虽未发现Cat X在4组中表达有差异，但Cat X水平高的结肠癌患者OS更短。Decock等^[19]^比较了早期乳癌和炎性乳癌中血清Cat X水平，发现炎性乳癌患者中血清Cat X中水平低，有可能为炎性乳癌的诊断提供一定的参考价值。本研究检测了肺癌患者和健康人血清Cat X水平，结果提示肺癌患者Cat X水平明显高于健康人，提示Cat X可能与肺癌发生有关，有希望作为肺癌的肿瘤标记物。

Cystatin C是Cats抑制剂，可能会减轻Cats在肿瘤侵袭、转移过程中作用^[20]^。Nishikawa等^[21]^用ELISA法检测良、恶性卵巢瘤患者和健康人的血清cystatin C，发现卵巢癌患者血清cystatin C水平明显高于良性肿瘤及健康人。Saleh等^[22]^也发现在结直肠癌组织中cystatin C高表达，特别是在腺癌中达到100%。然而也有研究^[23]^表明在肺鳞癌和正常肺组织中表达无明显差异。Wegiel等^[24]^则发现前列腺癌中cystatin C表达减低。我们检测了肺癌患者和健康人血清cystatin C水平，结果示肺癌患者血清cystatin C水平明显高于健康人。究其原因可能是肺癌患者中Cats升高，cystatin C为了抑制其活性也随之升高，但是仍然无法有效抑制蛋白水解酶的活性，导致肿瘤发生。

Cystatin C与Cats之间的失衡可能与肿瘤发生有关。Zore等^[25]^发现结肠癌患者血清Cat B的含量增加，cystatin C也随之增加，但Cat B的活性并未被相应增加的cystatin C抑制，提示随着肿瘤的进展，两者改变的同时伴随Cat B和cystatin C之间的失平衡。故有人把cystatin C与Cats的比值作为诊断肿瘤恶性程度的指标。本研究提示肺癌患者cystatin C/Cat X比值与健康人相比，无统计学差异，未见到cystatin C和Cat X失衡与肺癌相关。我们研究发现肺癌患者血清cystatin C和Cat X之间无相关性。Chen等^[26]^报道cystatin C与Cat B、Cat L之间无明显相关。原因可能是由于cystatin C并不是Cats唯一的抑制剂，其它抑制剂如stefn A也可能涉及到Cats的抑制。

本研究显示，Cat X与肺癌患者性别、年龄、病理类型、淋巴结转移、细胞分化及TNM分期之间无明显相关。Sevenich等^[27]^研究提示，在基因敲除的乳腺癌小鼠模型中，Cat X在肿瘤发展的初期表达增高，而在进展期及转移瘤中表达降低。此外Hidaka等^[13]^研究结果提示20q13.2扩增与结肠癌进展和转移有关。Wang等^[28]^研究也发现Cat X与晚期肝癌有关。而Lines等^[29]^报道了相反的结论，提示Cat X降低了细胞粘附，并减少胰腺癌转移。这些不同研究结果可能由于样本量偏小、检测方法以及检测标本不同或Cat X在不同肿瘤中表达不同所致。

本研究显示，cystatin C与肺癌分期有关，但是与其它临床特征之间无明显相关。Vigneswaran等^[30]^研究发现cystatin C与乳腺癌淋巴结转移无关，但与肿瘤大小有关。Saleh等^[22]^研究发现cystatin C在晚期结肠癌中表达高于早期结肠癌。本研究与上述研究一致，提示cystatin C升高可能与肿瘤浸润、转移有关。

本研究未发现cystatin C/Cat X比值与肺癌临床病理特征相关。Kolwijck等^[31]^报道cystatin C/Cat H和cystatin C/ Cat X比值与卵巢癌病理类型相关，在低分化癌中明显升高。cystatin C与Cat X失衡与肿瘤研究甚少，还需要进一步扩大样本量或在其它肿瘤上进一步研究。

本研究表明，Cat X低水平肺癌患者同高水平患者相比有更好的OS。Vizin等^[18]^报道了血清Cat X高水平的结肠癌患者OS更短，与结肠癌预后有关。本研究与Vizin等^[18]^研究一致，提示Cat X有希望作为判断肺癌预后的指标。

我们研究提示，血清cystatin C高水平的肺癌患者OS短于低水平者(22.6个月*vs* 13.7个月)，但差异无统计学意义(*P*=0.105)。Strojan等^[32]^研究报道cystatin C与头颈部肿瘤预后有关，cystatin C低表达的患者无病生存期更长。Kos等^[33]^研究了黑色素瘤患者中cystatin C与预后关系，未发现两者之间存在相关性。

我们用单因素*Cox*回归分析发现Cat X水平以及TNM分期是影响肺癌预后独立因素，Cat X高水平以及晚期肺癌患者预后更差。多因素回归分析表明分期是影响肺癌患者生存的独立危险因素，晚期患者预后更差、OS更短，死亡的风险是早期的2.250倍。我们的结果进一步佐证了晚期肺癌预后更差。

总之，我们的研究初步证明在肺癌患者中血清Cat X和cystatin C水平升高，可能与肺癌的发生、发展有关。cystatin C在晚期肺癌中表达增高，可能与肺癌转移、侵袭相关。Cat X高水平的患者OS更短，单因素*Cox*回归分析显示Cat X是影响肺癌患者预后的独立因素。但本研究样本量还不大，需要扩大样本量进一步研究Cat X等因子与肺癌临床特征及预后的关系。
